# Low Back Pain, Disability, and Quality of Life One Year following Intradiscal Injection of Autologous Bone Marrow Aspirate Concentrate

**DOI:** 10.1155/2022/9617511

**Published:** 2022-12-12

**Authors:** Colin M. Haines, Fenil R. Bhatt, Lindsay D. Orosz, Tarek Yamout, Samuel Namian, Niteesh Bharara, Anthony Bucci, Thomas C. Schuler, Ehsan Jazini, Christopher R. Good

**Affiliations:** ^1^Virginia Spine Institute, 11800 Sunrise Valley Drive, Suite 800, Reston, VA 20191, USA; ^2^National Spine Health Foundation, 11800 Sunrise Valley Drive, Suite 330, Reston, VA 20191, USA

## Abstract

**Introduction:**

Degenerative disc disease is a common cause of chronic low back pain. Surgical intervention is an invasive treatment associated with high costs. There is growing interest in regenerative medicine as a less invasive but direct disc treatment for chronic discogenic low back pain.

**Objective:**

To evaluate clinical improvement of primary discogenic low back pain with intradiscal injection of autologous bone marrow aspirate concentrate (BMAC). *Study Design*. Prospective cohort study. *Setting*. Single, multiphysician center. *Patients*. 32 adult patients undergoing intradiscal injection of autologous BMAC for the treatment of primary discogenic low back pain. *Interventions*. Intradiscal injection of autologous BMAC. *Main Outcome Measures*. Primary outcome measure is visual analog back pain scale (VAS back pain). Secondary outcome measures include ODI, VAS leg pain, and EQ-5D-5L scores. Outcomes were compared from baseline to 1 year.

**Results:**

Thirty-two patients (56.3% male) with a mean age of 45.9 years were enrolled, giving 92 treated levels. Mean VAS back and leg pain scores improved from 5.4 to 3.0 (*p* < 0.001) and 2.8 to 1.3 (*p* = 0.005), respectively. Mean ODI scores decreased from 33.5 to 21.1 (*p* < 0.001), and EQ-5D-5L scores improved from 0.69 to 0.78 (*p* = 0.001). Using established MCID values, 59.4% had clinically significant improvement in VAS back pain, 43.8% in VAS leg pain, and 56.3% in ODI scores.

**Conclusion:**

Intradiscal injection of autologous BMAC significantly improved low back pain, disability, and quality of life at one year. This study suggests that intradiscal BMAC has the potential to be an effective nonsurgical treatment for chronic discogenic low back pain.

## 1. Introduction

Chronic low back pain is one of the leading causes of disability in both the United States and worldwide, resulting in healthcare expenditures in the tens of billions of dollars [1–3]. Degenerative disc disease (DDD) has been well characterized as one of the primary etiologies of chronic low back pain [4]. The intervertebral discs are relatively avascular, resulting in a poor healing environment with a propensity to degenerate [5–7]. Surgical treatment is often medically appropriate for patients with symptomatic DDD who fail nonoperative treatment. While clinically effective, surgery is more invasive and with greater risks than nonoperative treatments [8]. There is growing interest in the field of regenerative medicine, including bone marrow aspirate intradiscal injections, as a less invasive treatment for low back pain [2, 9]. This direct treatment into damaged and painful discs offers a distinct advantage over (1) traditional nonoperative options that have an indirect effect on the discs and (2) surgical options that, while having a direct effect, can be associated with decreased lumbar range of motion and adjacent segment degeneration.

Regenerative medicine utilizes the body's cells to promote healing in an area of damage. Bone marrow aspirate concentrate (BMAC) has been shown to contain significant quantities of mesenchymal stem cells (MSC) and growth factors [10, 11]. As shown in animal models and other orthopedic applications, MSCs are pluripotent stem cells that can differentiate into the cell types that comprise cartilaginous tissues [2, 12–15]. The structural design of the intervertebral discs (inner nucleus pulposus and outer annulus fibrosis) makes the BMAC injection a promising adjunct to nonsurgical management of discogenic low back pain. There is a paucity of literature that reports on the clinical effectiveness of intradiscal BMAC injections. The purpose of this study is to investigate the potential to clinically improve chronic discogenic low back pain with an injection of autologous BMAC into degenerative lumbar discs through analysis of validated patient reported outcome measures (PROMs) at one year. The primary analysis will evaluate changes in low back pain from baseline to 1 year; secondary analyses will evaluate leg pain, disability rating, and quality of life scores from baseline to 1 year.

## 2. Methods

All literature searches were conducted using PubMed.

### 2.1. Patient Selection and Clinical Protocol

This is an IRB approved prospective cohort study conducted at a multiphysician spine practice. Adult patients (>18 years) diagnosed with primary discogenic low back pain, undergoing intradiscal injection of autologous BMAC from 2017 to 2019, and with PROMs completed at 1 year were eligible for inclusion. Patients were excluded if they (1) had an active infection, (2) were on chemotherapy, (3) were diagnosed with a myeloproliferative disorder, (4) were actively using nicotine, (5) were pregnant, and (6) did not complete PROMs. All patients signed informed consent and a Notice of Privacy Practices prior to the procedure.

Discogenic low back pain was diagnosed clinically through history, physical examination, and imaging, then confirmed diagnostically using lumbar discography. The subjective and objective clinical evaluation, discogram, and BMAC procedure were performed by the same physician per patient. In the rare case when lumbar discography was not available, pain provocation during disc pressurization at the start of the BMAC procedure was utilized as a surrogate confirmation of pain generators.

Subjectively, patients described low back pain with prolonged positioning made worse with bending, lifting, and/or sitting. Those with lower extremity complaints described these as minor compared to the predominant symptom of low back pain. For the objective evaluation, neurologic examination was normal or stable over the preceding 6 months of conservative treatments. All patients received standing lumbar anteroposterior, lateral, flexion, and extension X-rays and a lumbar MRI as part of their workup prior to the BMAC procedure. Findings such as overt instability, acute fractures, or major deformity were ruled out on X-ray. The Modic changes and Pfirrmann grading were obtained from the lumbar MRI, and findings such as severe stenosis, infection, and tumor were ruled out.

During lumbar discography, disc pain provocation was identified during disc pressurization with contrast dye injection per disc level, volumetric analysis was also measured with each injection, and dye patterns were assessed using live fluoroscopy during each injection. Subsequently, the patient's reported pain levels prior to and during the discogram, the quality of pain (concordant or nonconcordant), the quantity of dye in milliliters that the disc accepted, and the dye patterns were recorded for each injection per disc level. In this study, concordant pain was defined as the reproduction of the usual low back pain at baseline.

All patients underwent a minimum of 6 months of conservative treatment prior to consideration of BMAC injection. This included combinations of activity modification, exercise, physical therapy, chiropractic therapy, acupuncture, steroid injections, and medications (including NSAIDs, nonopioid pain relievers, muscle relaxers, neurolytics, and opioids). All BMAC procedures were performed in an outpatient facility.

### 2.2. Procedure Protocol

Under conscious sedation with intravenous Versed and fentanyl, subcutaneous injection of local anesthetic preceded bone marrow aspiration from each patient's posterior iliac crest utilizing fluoroscopic guidance while following standard sterile technique. Ten milliliter draws using a Jamshidi needle with repositioning between each draw were performed per the technique suggested by Hernigou et al. for a total of either 60 mL or 120 mL depending on the number of levels being treated [16]. Each syringe of aspirate was centrifuged through the Magellan autologous concentrate system (Isto Biologics, Hopkinton MA), and the amount of collected concentrate was recorded. The volume of concentrate ranged from 8 to 10 mL. Under fluoroscopic guidance, BMAC was injected into each symptomatic degenerative disc based on the accepted volume during prior discography and tactile sensation, ranging from 1 to 6 mL per disc level on average. These steps were all accomplished during a single procedure without modification to the concentrate.

### 2.3. Postprocedure Protocol

In accordance with the institution's postprocedure protocol, all patients were instructed to avoid high impact activities for 2 weeks, begin physical therapy approximately 2 weeks after the procedure, and avoid NSAID use for 6 weeks. Acetaminophen was recommended to use as needed for pain after the procedure, unless otherwise contraindicated. A prescription for an opioid pain reliever was provided based on individual patient needs.

### 2.4. Data Collection and Analysis

Basic demographics were obtained from electronic medical records and data capture systems. Questionnaires including Visual Analog Scale (VAS) for back and leg pain, Oswestry Disability Index (ODI), and EQ-5D-5L were administered at baseline and 1-year postprocedure to determine outcomes for back pain, leg pain, disability, and quality of life, respectively. Quantities of aspirated bone marrow and injected BMAC were individually measured and recorded during the procedure. Preprocedural MRIs were accessed through the institution's picture archiving and communication system (PACS) and reviewed by 2 fellowship trained spine surgeons. Each affected level was evaluated for Modic changes and assigned an original Pfirrmann grade [17, 18]. Postprocedure MRI was only performed if clinically indicated based on symptoms. When available, postprocedural MRIs were reviewed using the same methodology as the pre-preprocedural MRIs.

Statistical analyses were performed using IBM SPSS V 27.0 (Armonk, New York). Continuous variables are presented as means and standard deviations and categorical variables as counts and percentages. Comparison of PROM scores from baseline to 12 months was performed using paired *t*-tests; the level of significance was set at *p* < 0.05.

## 3. Results

### 3.1. Baseline Characteristics

Thirty-two patients (56.3% male), having a mean age of 45.9 ± 12.3 years, mean BMI of 27.1 ± 4.2 kg/m [2], and mean Charlson Comorbidity Index (CCI) score of 0.8 ± 1.0, were included (Table 1). On average, pain duration was 60.8 ± 15.8 months. The majority of patients (84.4%) underwent preprocedural discography of which 100% exhibited concordant back pain at a minimum of one disc level. All patients failed 6 months of nonoperative treatments, and 15 (45.0%) patients were taking an anti-inflammatory medication prior to the procedure. Mean preprocedural VAS back and leg pain scores were 5.4 ± 2.3 and 2.8 ± 2.5, respectively, on a scale of 0-10. Mean preprocedural ODI and EQ-5D-5L were 33.5 ± 13.6 and 0.7 ± 0.1, respectively (Table 2).

### 3.2. Procedure and Imaging

**Figure 1:**
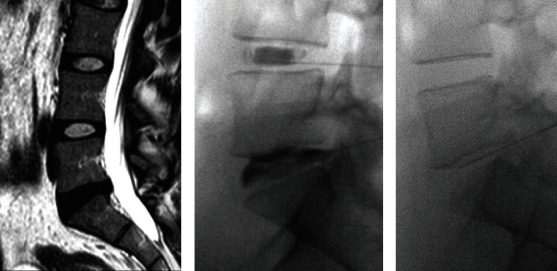
MRI showing disc desiccation at L5/S1 (a); discography showing abnormal dye pattern at L5/S1 (provoked concordant low back pain) and normal dye pattern at L4/5 (no pain provocation) (b); intradiscal BMAC injection at L5/S1 (c).

The total number of discs treated was 92 and ranged from 1 to 6 levels per patient with a mode of 3 levels being treated; the most treated levels were L4/5 (29.3%) and L5/S1 (30.4%). There were no procedural complications (Table 3). All patients had preprocedural MRI scans. On average, 62.5% of patients had Modic type 1 or 2 changes (40.2% per level) and 93.8% had a Pfirrmann grade of 3 or higher (90.2% per level). Postprocedural MRIs were obtained on 13 (40.6%) patients, of which 69.2% had no measurable change in the Pfirrmann grade. Three patients worsened, and one improved by one Pfirrmann grade (Table 4).  Figure 1 demonstrates a preprocedural MRI, fluoroscopic image from lumbar discography, and fluoroscopic image during the BMAC injection.

### 3.3. Outcomes

**Figure 2:**
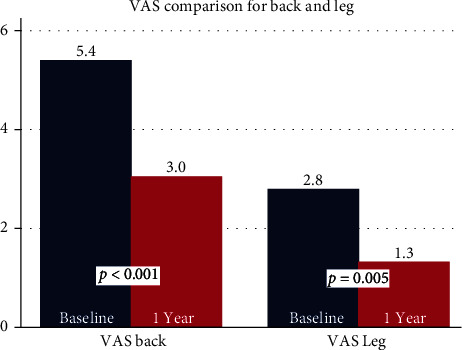
Bar graph comparing mean VAS scores at baseline and 1 year: back (left) and leg (right).

**Figure 3:**
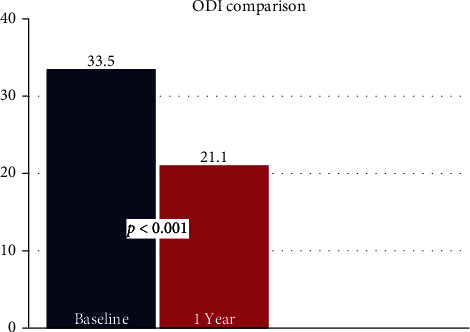
Bar graph comparing mean ODI scores at baseline and 1 year.

**Figure 4:**
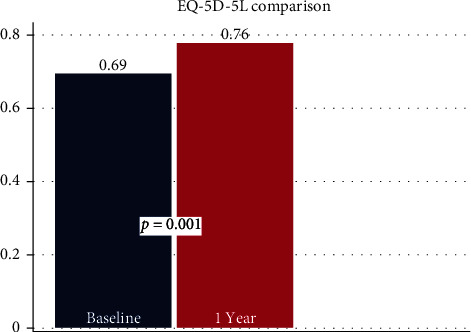
Bar graph comparing mean EQ-5D-5L scores at baseline and 1 year.

Mean VAS back pain scores decreased by 2.4 points (*p* < 0.001) from pre- to 1-year postprocedure. VAS leg pain scores decreased by 1.5 points (*p* = 0.005) (Figure 2). There was a decrease of 12.4 points in mean ODI score (*p* < 0.001) (Figure 3). Mean EQ-5D-5L improved by 0.09 points (*p* = 0.001) (Figure 4). Overall, 59.4% of patients achieved a clinically significant improvement in VAS back pain, 43.8% in VAS leg pain, and 56.3% in ODI scores per established minimum clinically important difference (MCID) values (Table 5) [19].

There were no complications reported relating to the injection within one-year follow-up. Three patients (9.4%) went on to have fusion surgery at levels previously treated with BMAC due to persistent pain at an average of 164.5 days post injection (Table 6).

## 4. Discussion

The results of this study show significant improvements in VAS back pain, VAS leg pain, ODI, and EQ-5D-5L at one-year follow-up when compared to baseline values for patients treated with intradiscal injection of autologous BMAC for discogenic low back pain. Clinically, significant improvements exceeding established MCID values in VAS back pain (59.4%), VAS leg pain (43.8%), and ODI (56.3%) were found [19]. Despite a cohort of relatively young and healthy nonsmokers, the baseline PROMs are indicative of patients with moderate disability [20] .

The improvement in VAS leg scores was unexpected but has been reported once previously in the literature [13]. While an exact reason for this is unknown, this may be the result of morphologic improvements in disc structure or the anti-inflammatory effects of BMAC injection contributing to reduced nerve irritation, which has been previously characterized as an effect of mesenchymal stem cells [21, 22].

Early systematic and comprehensive reviews found that there are limited studies describing validated patient reported outcome measures following intradiscal BMAC injection and the overall quality of evidence was low [4, 23]. However, more recent reviews have demonstrated that there is an increasing body of literature, including randomized controlled trials (RCTs), which describe improved patient reported outcomes as a result of intradiscal BMAC injection [24].

While early research suggested that intradiscal BMAC injection does not improve low back pain, subsequent studies have shown promising results [13, 25, 26]. A retrospective study by Wolff et al. on a cohort of 33 patients who received intradiscal BMAC injections demonstrated improvements in lower back pain based on numeric rating scale, ODI, and SF-26 scores. All three outcomes improved by at least 50% in over 30% of the patients [27].

The most compelling prospective data using standardized outcome measures on this topic are a series of papers published by Pettine et al. on a cohort of 26 patients. These papers were published with follow-up periods of 1, 2, 3, and 5 years [28–31]. In their 12-month follow-up, they reported an average decrease of VAS back pain score by 46.1 points (on a 0-100 scale) and ODI score by 31.5 points [28]. Our study also demonstrated a decrease of VAS back pain and ODI scores in all patients at 12 months after BMAC injection, with an average reduction of 2.4 (on a 0-10 scale) and 12.4 points, respectively. In Pettine et al.'s study, patients that did not have at least a 25% improvement in pain by the 6-month follow-up were eligible for reinjection of the affected levels. This contrasts with our study, where patients were injected only once.

In another prospective study, Elabd et al. evaluated patient reported outcomes using a percentage of overall improvement (0-100% scale) 4-6 years following autologous intradiscal hypoxic cultured BMAC injection in five patients. They found that improvement ranged from 10 to 90%, with a mean improvement of 55%. All 5 patients reported that strength was improved, and 4 out of 5 patients reported that mobility was also improved [32].

In an RCT, Noriega et al. evaluated 24 patients who were randomized into two groups, with the test group receiving allogeneic BMAC and the control group receiving a placebo infiltration of the paravertebral musculature with anesthetic. Of note, this study utilized allogeneic BMAC unlike our study which utilized autologous BMAC. Patient-reported outcomes were collected using the VAS pain scale, ODI, and SF-12 life quality questionnaires coupled with MRI exploration throughout the course of 6 follow-up visits over 12 months. Patients in the treatment arm displayed a significant improvement of 28% with their VAS scores (*p* < 0.001) as opposed to the control group which did not exhibit statistically significant improvement in this category. Furthermore, the quantified Pfirrmann grading showed a small but statistically significant improvement in the treated patients while the opposite was found in the control group on MRI at 1 year [33].

In the literature, MRIs have been similarly reviewed before and after BMAC with results varying from no changes in disc morphology to modest improvements in disc height [26, 28]. MRIs in our cohort were analyzed using the original Pfirrmann classification system and Modic endplate changes [17, 18]. The majority had Pfirrmann grades 3-5 (93.8% preprocedural and 100% postprocedural) and Modic 1-2 changes (62.5% preprocedural and 69.2% postprocedural). Comparing pre- and postprocedure MRIs, 69.2% had no measurable change in the Pfirrmann grade, three (23.1%) patients worsened, and one (7.7%) improved by the one Pfirrmann grade. This suggests that greater importance should be placed on PROMs in the evaluation of patient response to treatment rather than MRI findings.

MRI analysis has been performed as part of evaluating the clinical efficacy of other regenerative options for the treatment of low back pain, such as platelet-rich plasma (PRP). At the 52-week follow-up, Murray et al.'s intradiscal PRP releasate group had clinically significant improvements in their VAS and ODI scores from baseline (−53.4 ± 24.7 and −26.6 ± 14.8, respectively). On MRI analysis at 52 weeks, there was no change in the Pfirrmann or modified Pfirrmann grading scores compared to baseline, similar to the findings of this study [34].

Intradiscal BMAC injection is less invasive than surgery; however, surgery can be used as the next treatment option if BMAC injection fails to adequately improve symptoms. There is limited data reported on the number of patients who elect to undergo surgery following an intradiscal BMAC injection. Within the 1-year follow-up period of this study, 3 (9.4%) patients elected to undergo fusion surgery due to continued pain at their affected levels. In the similar study by Pettine et al., 2 (7.7%) patients elected to have a surgical procedure by the 1-year follow-up [31].

### 4.1. Strengths and Limitations

The American Academy of Orthopaedic Surgeons published reporting standards for studies relating to platelet-rich plasma and mesenchymal stem cells due to prior inconsistencies in result reporting to help guide future therapy. This study meets 88% of AAOS reporting standards and is a compelling addition to the current literature [34].

Limitations of this study include small sample size and limited follow-up period. While we were able to report statistical improvements in PROMs, long-term changes in these scores beyond one year are unknown. Additional studies with longer follow-up periods and larger sample sizes are needed. Further prospective randomized studies with comparison cohorts are warranted to further evaluate the efficacy of BMAC. The results of this study can serve as a precursor to larger trials. The authors intend to investigate this topic further with larger sample sizes, longer duration of follow-up, and in-depth subanalyses to better account for potential confounders.

We did not inject a standardized amount of BMAC per level. The quantity injected was based on the volume accepted per disc and ranged from 1 to 6 mL per level. As such, we cannot draw any conclusions regarding any effects between quantity injected and effect on pain. The BMAC was not cultured or analyzed; no conclusions can be made about the relative stem cell concentrations of each patient's BMAC. Future studies that standardize this portion of the methodology could reduce any possible sources of error in the clinical procedure and further elucidate the relationship between microbiological characteristics of aspirate and clinical outcome.

## 5. Conclusion

This study suggests that autologous intradiscal BMAC injection has the potential to clinically improve discogenic low back pain at one year while reducing disability ratings and increasing quality of life scores. This regenerative medicine procedure offers a direct and promising adjunct to traditional nonoperative treatments for chronic discogenic low back pain.

## Figures and Tables

**Table 1:** Patient characteristics.

Characteristic	Value
*N*	32
Male sex	18 (56.3)
Mean age (years)	45.9 ± 12.3
Mean CCI score	0.8 ± 1.0
Mean BMI (kg/m^2^)	27.1 ± 4.2
Nicotine use	0 (0.0)

Values represent the number of patients (%) or mean ± SD.

**Table 2:** Preprocedural details.

Variable	Value
Mean pain duration (months)	60.8 ± 15.8
Axial back pain	32 (100.0)
Radiculopathy	18 (56.3)
Mean preop VAS back (0-10)	5.4 ± 2.3
Mean preop VAS leg (0-10)	2.8 ± 2.5
Mean preop ODI	33.5 ± 13.6
Mean preop EQ-5D-5L	0.7 ± 0.1
Discography obtained	27 (84.4)
Concordant back pain	27 (100.0)
Prior decompression at treated or adjacent level	9 (28.1)

Values represent the number of patients (%) or mean ± SD.

**Table 3:** Procedural details.

Variable	Value
No. disc levels treated	92
T12/L1	3 (3.3)
L1/2	4 (4.3)
L2/3	12 (13.0)
L3/4	18 (19.6)
L4/5	27 (29.3)
L5/S1	28 (30.4)
No. disc levels treated per patient	32
1-2 levels	13 (40.6)
3-4 levels	15 (46.9)
5-6 levels	4 (12.5)
Total aspirate (mL)	
60	22 (68.8)
120	10 (31.3)
Total concentrate (mL)	10.5 ± 5.5
Injected per level (mL)	3 ± 0.4
Complications	0 (0.0)

Values represent the number of patients (%) or mean ± SD. No.: number.

**Table 4:** Disc evaluation.

Variable	Value	
	Per patient	Per level
Preprocedure MRI		
Total	32	92
No Modic changes	12 (37.5)	55 (59.8)
Modic 1-2	20 (62.5)	37 (40.2)
Modic 3	0 (0.0)	0 (0.0)
Pfirrmann grades 1-2	2 (6.3)	9 (9.8)
Pfirrmann grades 3-5	30 (93.8)	83 (90.2)
Postprocedure MRI		
Total	13 (40.6)	40 (43.5)
No Modic changes	4 (30.8)	26 (65.0)
Modic 1-2	9 (69.2)	14 (35)
Modic 3	0 (0.0)	0 (0.0)
Pfirrmann grades 1-2	0 (0.0)	4 (10.0)
Pfirrmann grades 3-5	13 (100)	36 (90.0)

Values represent the number of patients (%) or mean ± SD. MRI: magnetic resonance imaging.

**Table 5:** Clinically important 1-year results.

Outcome measure	Established MCID Value	Patients Exceeding MCID
ODI	>12.8	18 (56.3)
VAS back	>12	19 (59.4)
VAS leg	>16	14 (43.8)

Values represent the number of patients (%). MCID: minimum clinically important difference; ODI: Oswestry Disability Index; VAS: Visual Analog Scale.

**Table 6:** Postprocedure surgery details.

Variable	Value
Subsequent fusion surgery	3 (9.4)
Average time to surgery (days)	164.5 ± 29.0

Values represent the number of patients (%) or mean ± SD.

## Data Availability

Access to data is restricted due to patient privacy practices.
